# Crystal structure and Hirshfeld surface analysis of dimethyl 4′-bromo-3-oxo-5-(thio­phen-2-yl)-3,4,5,6-tetra­hydro-[1,1′-biphen­yl]-2,4-di­carboxyl­ate

**DOI:** 10.1107/S2056989024002858

**Published:** 2024-04-04

**Authors:** Farid N. Naghiyev, Victor N. Khrustalev, Mehmet Akkurt, Khammed A. Asadov, Ajaya Bhattarai, Ali N. Khalilov, İbrahim G. Mamedov

**Affiliations:** a Department of Chemistry, Baku State University, Z. Khalilov str. 23, Az, 1148, Baku, Azerbaijan; b Peoples’ Friendship University of Russia (RUDN University), Miklukho-Maklay St. 6, Moscow, 117198, Russian Federation; c N. D. Zelinsky Institute of Organic Chemistry RAS, Leninsky Prosp. 47, Moscow, 119991, Russian Federation; d Department of Physics, Faculty of Sciences, Erciyes University, 38039 Kayseri, Türkiye; e Department of Chemistry, M.M.A.M.C (Tribhuvan University) Biratnagar, Nepal; f "Composite Materials" Scientific Research Center, Azerbaijan State Economic University (UNEC), Murtuza Mukhtarov str. 194, Az 1065, Baku, Azerbaijan; Institute of Chemistry, Chinese Academy of Sciences

**Keywords:** crystal structure, disorder, C—H⋯S hydrogen bonds, cyclo­hexene ring, thio­phene ring, Hirshfeld surface analysis

## Abstract

In the title compound, C_20_H_17_BrO_5_S, mol­ecules are connected by inter­molecular C—H⋯S hydrogen bonds with *R* ^2^_2_(10) ring motifs, forming ribbons along the *b*-axis direction. C—H⋯π inter­actions consolidate the ribbon structure while van der Waals forces between the ribbons ensure the cohesion of the crystal structure. According to a Hirshfeld surface analysis, H⋯H (40.5%), O⋯H/H⋯O (27.0%), C⋯H/H⋯C (13.9%) and Br⋯H/H⋯Br (11.7%) inter­actions are the most significant contributors to the crystal packing. The thio­phene ring and its adjacent di­carboxyl­ate group and the three adjacent carbon atoms of the central hexene ring to which they are attached were refined as disordered over two sets of sites having occupancies of 0.8378 (15) and 0.1622 (15). The thio­phene group is disordered by a rotation of 180° around one bond.

## Abstract 2

In the title compound, mol­ecules are connected by inter­molecular C—H⋯S hydrogen bonds with 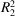 (10) ring motifs, forming ribbons along the *b*-axis direction. C—H⋯π inter­actions consolidate the ribbon structure while van der Waals forces between the ribbons ensure the cohesion of the crystal structure.

## 1. Chemical context

Functionalized carbo- and heterocyclic compounds are important systems in different fields of science (Huseynov *et al.*, 2023 ▸; Akkurt *et al.*, 2023 ▸). These systems comprise nucleic acids, alkaloids, vitamins, sugars, hormones, anti­biotics, other drugs, dyes, pesticides, and herbicides. There have been crucial developments in organic synthesis with heterocyclic systems designed recently for various research and commercial purposes (Maharramov *et al.*, 2022 ▸; Erenler *et al.*, 2022 ▸, Khalilov *et al.*, 2023 **a* ▸,b* ▸). These derivatives have found widespread applications in coordination (Gurbanov *et al.*, 2021 ▸; Mahmoudi *et al.*, 2021 ▸), medicinal (Askerova, 2022 ▸) and materials chemistry (Velásquez *et al.*, 2019 ▸; Afkhami *et al.*, 2019 ▸). These ring systems are used for a large range of applications, as well as drugs, ligands, catalysts, and materials (Maharramov *et al.*, 2021 ▸, Sobhi & Faisal, 2023 ▸). Functionalized systems combining cyclo­hexa­none, phenyl and thio­phene motifs exhibit various biological activities, such as molluscicidal, anti­cancer, anti­oxidant, cytotoxic, anti-inflammatory, herbicidal, pesticidal, and anti­bacterial (Atalay *et al.*, 2022 ▸; Donmez & Turkyılmaz, 2022 ▸). As a result of the varied applications of these systems, their efficient and regioselective development has attracted great attention. Thus, in the framework of our structural studies (Abdinov *et al.*, 2004 ▸, 2012 ▸, 2014 ▸; Naghiyev *et al.*, 2021*b* ▸), herein we report the crystal structure and Hirshfeld surface analysis of the title compound, dimethyl 4′-bromo-3-oxo-5-(thio­phen-2-yl)-3,4,5,6-tetra­hydro-[1,1′-biphen­yl]-2,4-di­carboxyl­ate. 
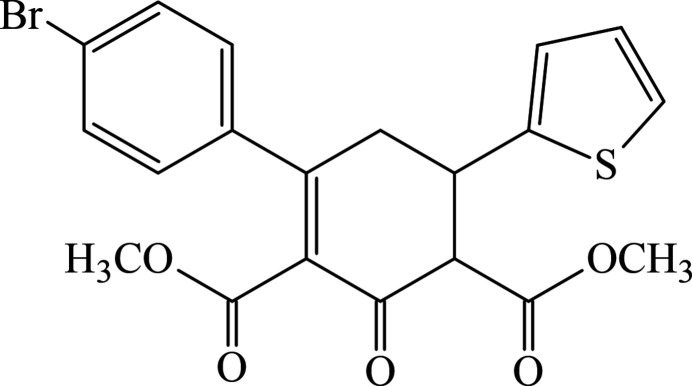


## 2. Structural commentary

**Figure 1:**
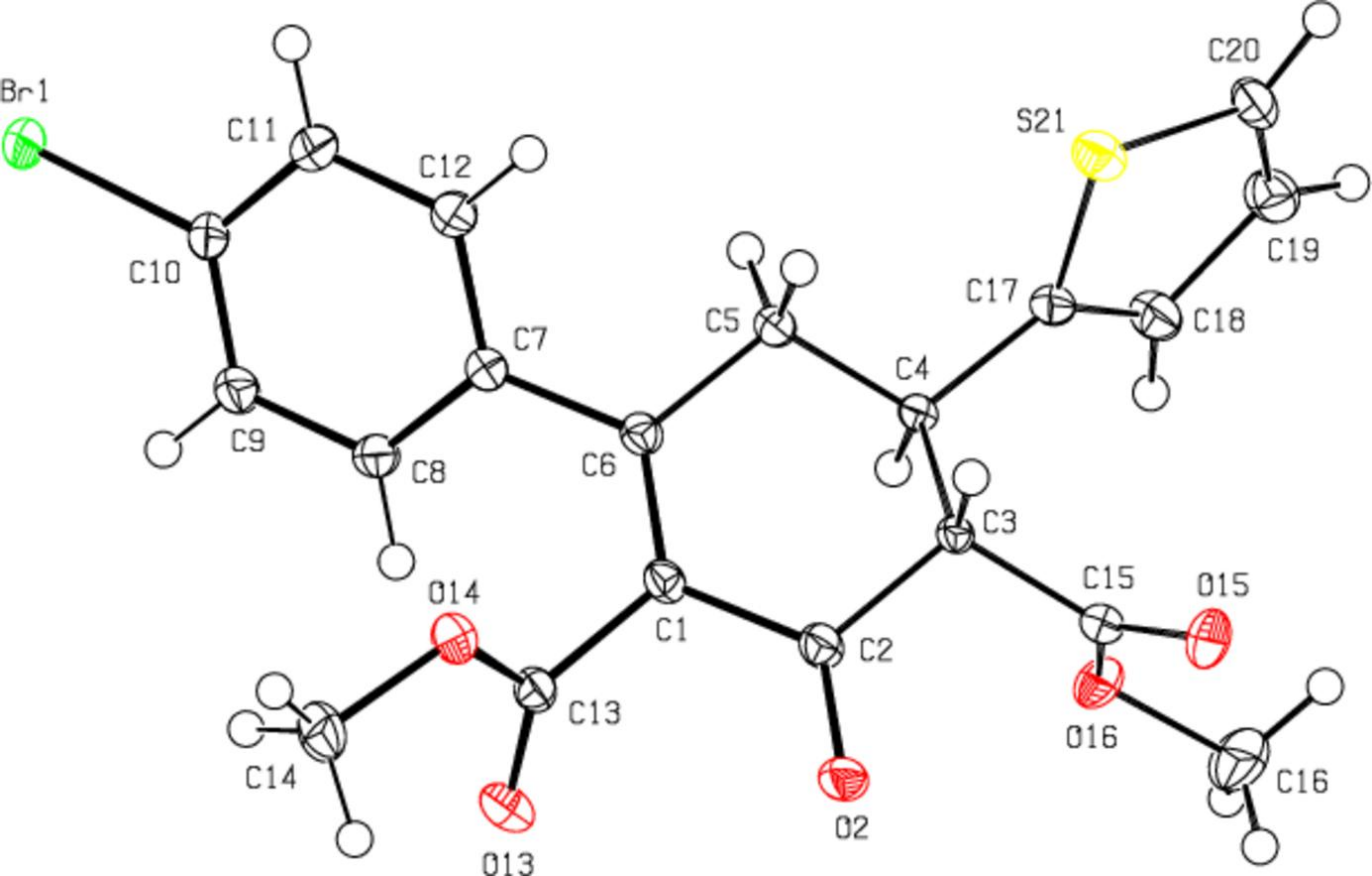
The mol­ecular structure of the title compound, showing the atom labelling and displacement ellipsoids drawn at the 50% probability level.

As seen in Fig. 1 ▸, the major (C1–C6) component of the central hexene ring shows a distorted boat conformation [puckering parameters (Cremer & Pople, 1975 ▸) are *Q*_T_ = 0.5077 (16) Å, θ = 129.02 (17)°, φ = 355.7 (2)°], and the minor (C1/C2/C3*A–*C5*A/*C6) component of the central hexene ring also shows an envelope conformation [puckering parameters *Q*_T_ = 0.568 (7) Å, θ = 54.8 (5)°, φ = 124.2 (6)°]. The r.m.s planes of these disordered hexene rings make angles of 72.18 (14), 69.6 (9), 49.52 (7), and 62.5 (2), 60.1 (9), 44.23 (17), respectively, with the major (S21/C17–C20) and minor (S21*A*/C17*A*–C20*A*) disordered thio­phene ring components and the benzene ring (C7–C12). The C2—C1—C13—O13, C1—C13—O14—C14, C2—C3—C15—O15, C2—C3*A*—C15*A*—O15*A*, C3—C15—O16—C16 and C3*A*—C15*A*—O16*A*—C16*A* torsion angles are −64.22 (19), 177.23 (12), −107.7 (4), −64 (3), 175.03 (18) and 177.9 (11)°, respectively. The geometric parameters of the title compound are normal and comparable to those of related compounds listed in the *Database survey* section.

## 3. Supra­molecular features and Hirshfeld surface analysis

**Figure 2:**
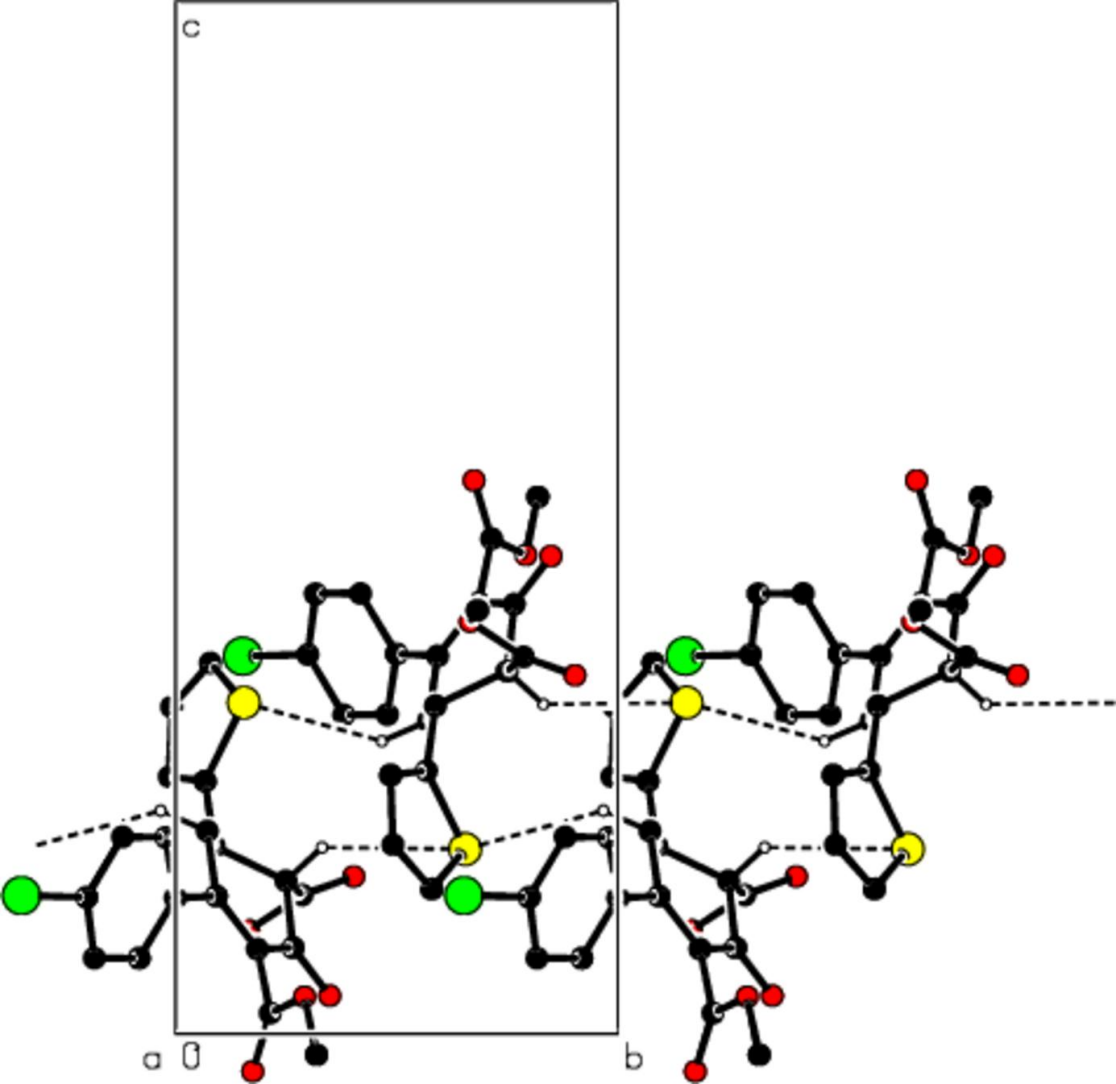
The packing viewed down the *a* axis of the title compound with C—H⋯S hydrogen bonds shown as dashed lines.

**Figure 4:**
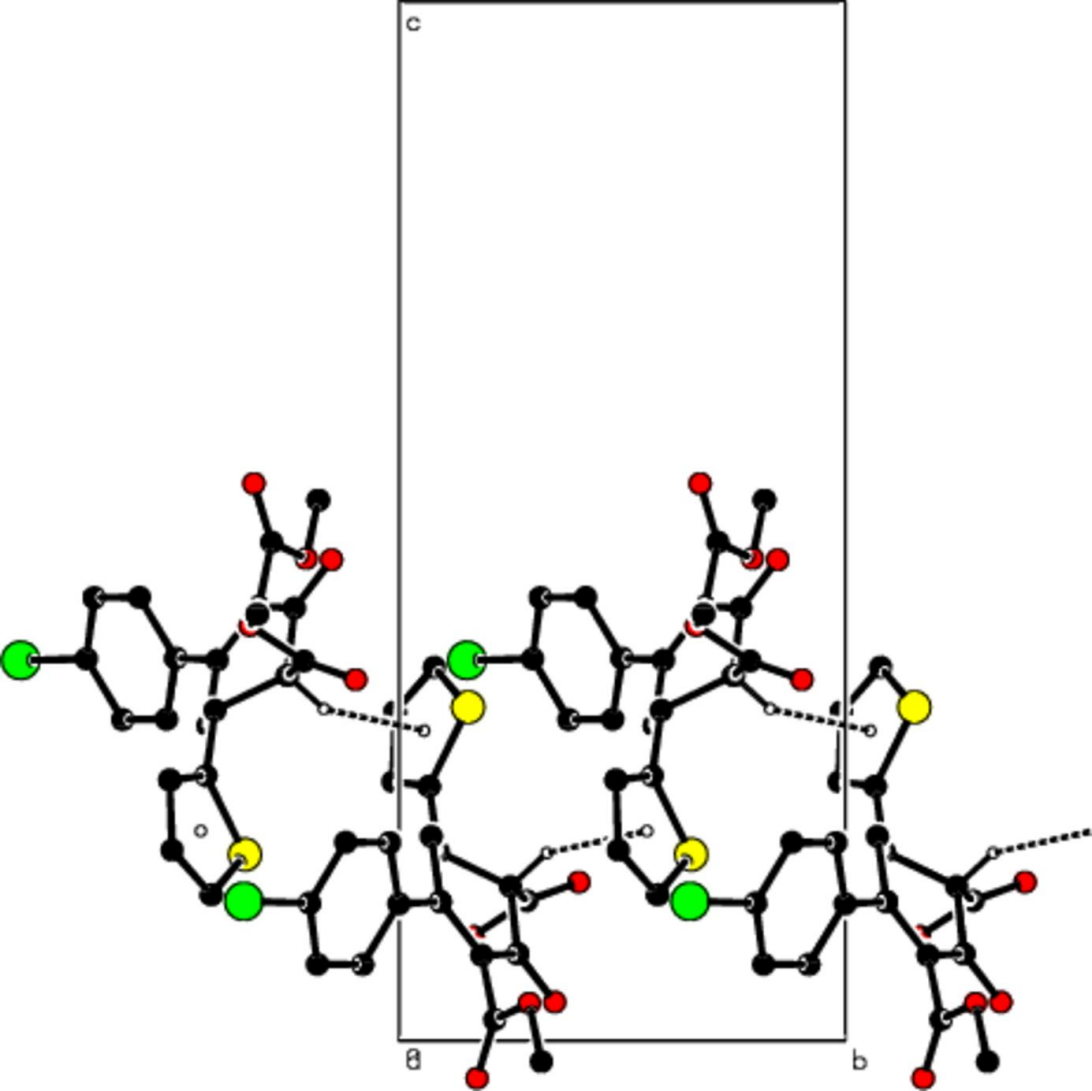
A view of the packing down the *a* axis of the title compound with C—H⋯π inter­actions shown as dashed lines.

In the crystal, mol­ecules are connected by inter­molecular C—H⋯S hydrogen bonds with 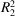 (10) ring motifs (Table 1 ▸; Figs. 2 ▸ and 3 ▸; Bernstein *et al.*, 1995 ▸), forming ribbons along the *b*-axis direction. C—H⋯π inter­actions consolidate the ribbon structure while van der Waals forces between the ribbons ensure the cohesion of the crystal structure (Table 1 ▸; Figs. 4 ▸ and 5 ▸).

**Figure 6:**
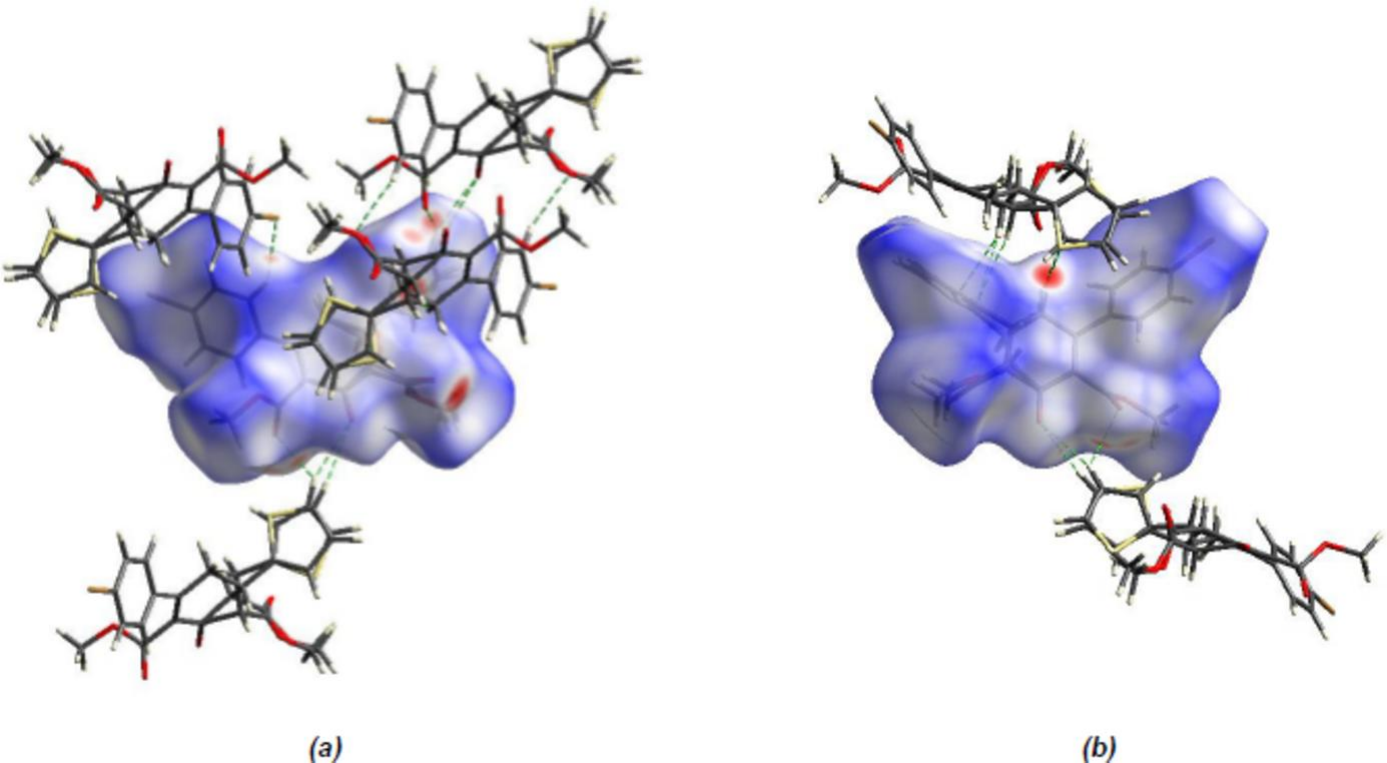
(*a*) Front and (*b*) back sides of the three-dimensional Hirshfeld surface of the title compound mapped over *d_norm_*.

To qu­antify the inter­molecular inter­actions between the mol­ecules in the crystal structure of the title compound, a Hirshfeld surface analysis was performed and the two-dimensional fingerprint plots generated using *CrystalExplorer 17.5* (Spackman *et al.*, 2021 ▸). The Hirshfeld surfaces were mapped over *d*_norm_ in the range −0.2669 (red) to +1.2638 (blue) a.u. (Fig. 6 ▸).

**Figure 7:**
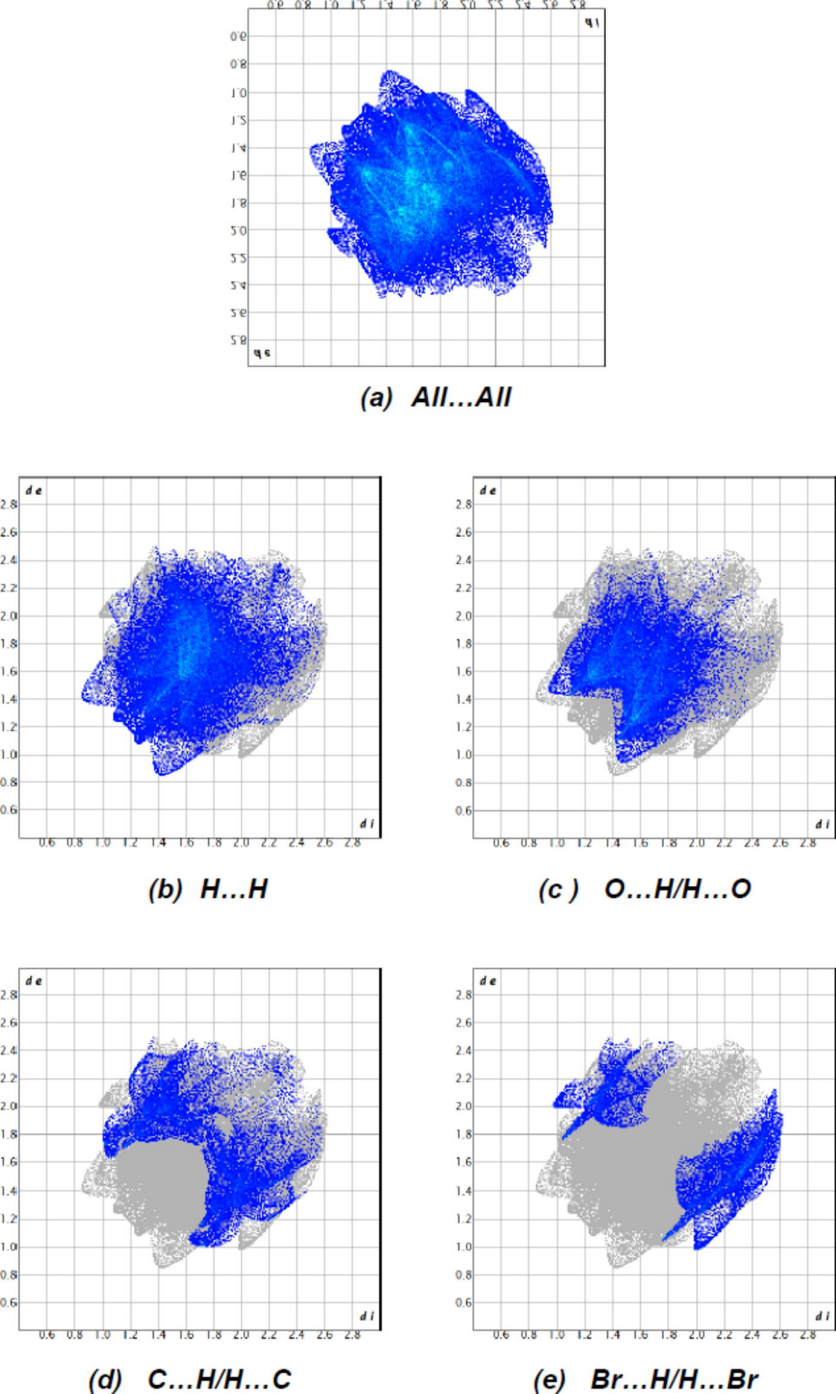
The two-dimensional fingerprint plots, showing (*a*) all inter­actions, and delineated into (*b*) H⋯H, (*c*) O⋯H/H⋯O, (*d*) C⋯H/H⋯C and (*e*) Br⋯H/H⋯Br inter­actions [*d*_e_ and *d*_i_ represent the distances from a point on the Hirshfeld surface to the nearest atoms outside (external) and inside (inter­nal) the surface, respectively].

The dominant inter­atomic contact is H⋯H as it makes the highest contribution to the crystal packing (40.5%, Fig. 7 ▸ *b*). Other major contributors are O⋯H/H⋯O (27.0%, Fig. 7 ▸ *c*), Br⋯H/H⋯Br (11.7%, Fig. 7 ▸ *d*) and C⋯H/H⋯C (13.9%, Fig. 7 ▸ *e*) inter­actions. Other, smaller contributions are made by C⋯C (2.9%), Br⋯O/O⋯Br (1.7%), S⋯H/H⋯S (1.2%), O⋯C/C⋯O (0.8%), O⋯O (0.2%) and S⋯C/C⋯S (0.1%) inter­actions.

## 4. Database survey

A search of the Cambridge Structural Database (CSD, Version 5.43, last update November 2022; Groom *et al.*, 2016 ▸) for the central six-membered *cyclo­hexene* ring yielded eight compounds related to the title compound, *viz*. CSD refcodes UPOMOE (Naghiyev *et al.*, 2021*a* ▸), ZOMDUD (Gein *et al.*, 2019 ▸), PEWJUZ (Fatahpour *et al.*, 2018 ▸), OZUKAX (Tkachenko *et al.*, 2014 ▸), IFUDOD (Gein *et al.*, 2007 ▸), IWEVOV (Mohan *et al.*, 2003 ▸), IWEVUB (Mohan *et al.*, 2003 ▸) and HALROB (Ravikumar & Mehdi, 1993 ▸).

UPOMOE and ZOMDUD crystallize in the monoclinic space group *P*2_1_/*c*, with *Z* = 4, PEWJUZ in *I*2/*c* with *Z* = 4, IFUDOD, HALROB and IWEVUB in *P*2_1_/*n* with *Z* = 4, and IWEVOV and OZUKAX in the ortho­rhom­bic space group *Pbca* with *Z* = 8. In UPOMOE, the central cyclo­hexane ring adopts a chair conformation. In the crystal, mol­ecules are linked by N—H⋯O, C—H⋯O, and C—H⋯N hydrogen bonds, forming mol­ecular layers parallel to the *bc* plane, which inter­act by the van der Waals forces between them. In ZOMDUD, mol­ecules are linked by inter­molecular N—H⋯O and C—H⋯O hydrogen bonds, forming a three-dimensional network. C—H⋯π inter­actions are also observed. In PEWJUZ, mol­ecules are linked by inter­molecular N—H⋯O and C—H⋯O hydrogen bonds, forming sheets parallel to the *bc* plane. C—H⋯π inter­actions are also observed. In OZUKAX, mol­ecules are linked by inter­molecular N—H⋯O and C—H⋯O hydrogen bonds, forming sheets parallel to the *ac* plane. C—H⋯π inter­actions are also observed. Inter­molecular O—H⋯O hydrogen bonds consolidate the mol­ecular conformation. There are no classical hydrogen bonds in the crystal of IFUDOD where inter­molecular C—H⋯O contacts and weak C—H⋯π inter­actions lead to the formation of a three-dimensional network. In the crystal of IWEVOV, the mol­ecules pack such that both carbonyl O atoms participate in hydrogen-bond formation with symmetry-related amide nitro­gen atoms present in the carbamoyl substituents, forming N—H⋯O hydrogen bonds in a helical arrangement. In the crystal, the phenyl rings are positioned so as to favour edge-to-edge aromatic stacking. When the crystal packing is viewed normal to the *ac* plane, it reveals a ’wire-mesh’ type hydrogen-bond network. In the crystal of IWEVUB, unlike in IWEVOV where both carbonyl O atoms participate in hydrogen bonding, only one of the carbonyl oxygen atoms participates in inter­molecular N—H⋯O hydrogen bonding while the other carbonyl oxygen participates in a weak C—H⋯O inter­action. In addition, one of the amide nitro­gen atoms participates in N—H⋯O hydrogen bonding with the hydroxyl oxygen atom, linking the mol­ecules in a helical arrangement, which is similar to that in the structure of IWEVOV. As observed in the structure of IWEVOV, the packing of the mol­ecules viewed normal to the *ab* plane resembles a ’wire-mesh’ arrangement of the mol­ecules. In the crystal of HALROB, the amide carbonyl groups are oriented in different directions with respect to the cyclo­hexa­none ring. These orientations of the carboxamide groups facilitate the formation of an intra­molecular O—H⋯O hydrogen bond. The mol­ecules are packed such that chains are formed along the *b*-axis direction. These chains are held together by N—H⋯O hydrogen bonds.

## 5. Synthesis and crystallization

A solution of 1-(4-bromo­phen­yl)-3-(thio­phen-2-yl)prop-2-en-1-one (5.2 mmol) and dimethyl-1,3-acetonedi­carboxyl­ate (5.2 mmol) in methanol (30 mL) was stirred for 10 min. Then *N*-methyl­piperazine (3 drops) was added to the reaction mixture, which was heated for 20 minutes at 318–323 K and stirred for 48 h at room temperature. Then 20 mL of methanol were removed from the reaction mixture, which was left overnight.

The precipitated crystals were separated by filtration and recrystallized from an ethanol/water (1:1) solution (m.p. = 508–509 K, yield 79%).

^1^H NMR (300 MHz, DMSO-*d*_6_, ppm., *J_HH_*, Hz): 3.04 (*d*, 2H, CH_2_, ^3^ *J*_H–H_ = 7.9); 3.52 (*k*, 1H, CH, ^3^ *J*_H–H_ = 7.9); 3.57 (*s*, 6H, 2OCH_3_); 4.15 (*d*, 1H, CH, ^3^ *J*_H–H_ = 8.7); 6.98 (*t*, 1H, CH_thien._,^3^ *J*_H–H_ = 5.1); 7.05 (*d*, 1H, CH_thien._,^3^ *J*_H–H_ = 5.1); 7.40 (*m*, 3H, 2CH_arom._ + CH_thien._); 7.67 (*d*, 2H, 2CH_arom._,^3^ *J*_H–H_ = 8.1). ^13^C NMR (75 MHz, DMSO-*d*_6_, ppm): 38.16 (CH), 38.25 (CH_2_), 52.43 (OCH_3_), 52.64 (OCH_3_), 60.15 (CH), 124.08 (C_arom._), 125.21 (CH_thien._), 125.51 (CH_thien._), 127.44 (CH_thien._), 129.30 (2CH_arom._), 131.62 (C_thien._), 132.22 (2CH_arom._), 137.40 (C_arom._), 144.19 (C_quat._), 159.21 (C_quat._), 166.37 (CO), 169.47 (CO), 190.91 (C=O).

## 6. Refinement

Crystal data, data collection and structure refinement details are summarized in Table 2 ▸. All H atoms were placed in calculated positions (C—H = 0.95–1.00 Å) and refined as riding with *U*_iso_(H) = 1.2*U*_eq_(N) or 1.5*U*_eq_(C). The thio­phene ring (S21/C17–C20) and its adjacent di­carboxyl­ate group (C15–C16/O15/O16) and the three adjacent carbon atoms (C3, C4 and C5) of the central hexane ring to which they are attached were refined as disordered over two sets of atomic sites having occupancies of 0.8378 (15) and 0.1622 (15). The methyl­ene carbon atom (C5) of the hexane ring was also refined with the same occupation ratio [0.8378 (15): 0.1622 (15)], having two disordered parts at the same position and the same displacement parameters using the EXYZ and EADP commands. The thio­phene group is disordered by a rotation of 180° around one bond. SADI, DFIX and EADP commands were used in the refinement.

## Supplementary Material

Crystal structure: contains datablock(s) I. DOI: 10.1107/S2056989024002858/nx2007sup1.cif

Structure factors: contains datablock(s) I. DOI: 10.1107/S2056989024002858/nx2007Isup2.hkl

Supporting information file. DOI: 10.1107/S2056989024002858/nx2007Isup3.cml

CCDC reference: 2344995

Additional supporting information:  crystallographic information; 3D view; checkCIF report

## Figures and Tables

**Figure 3:**
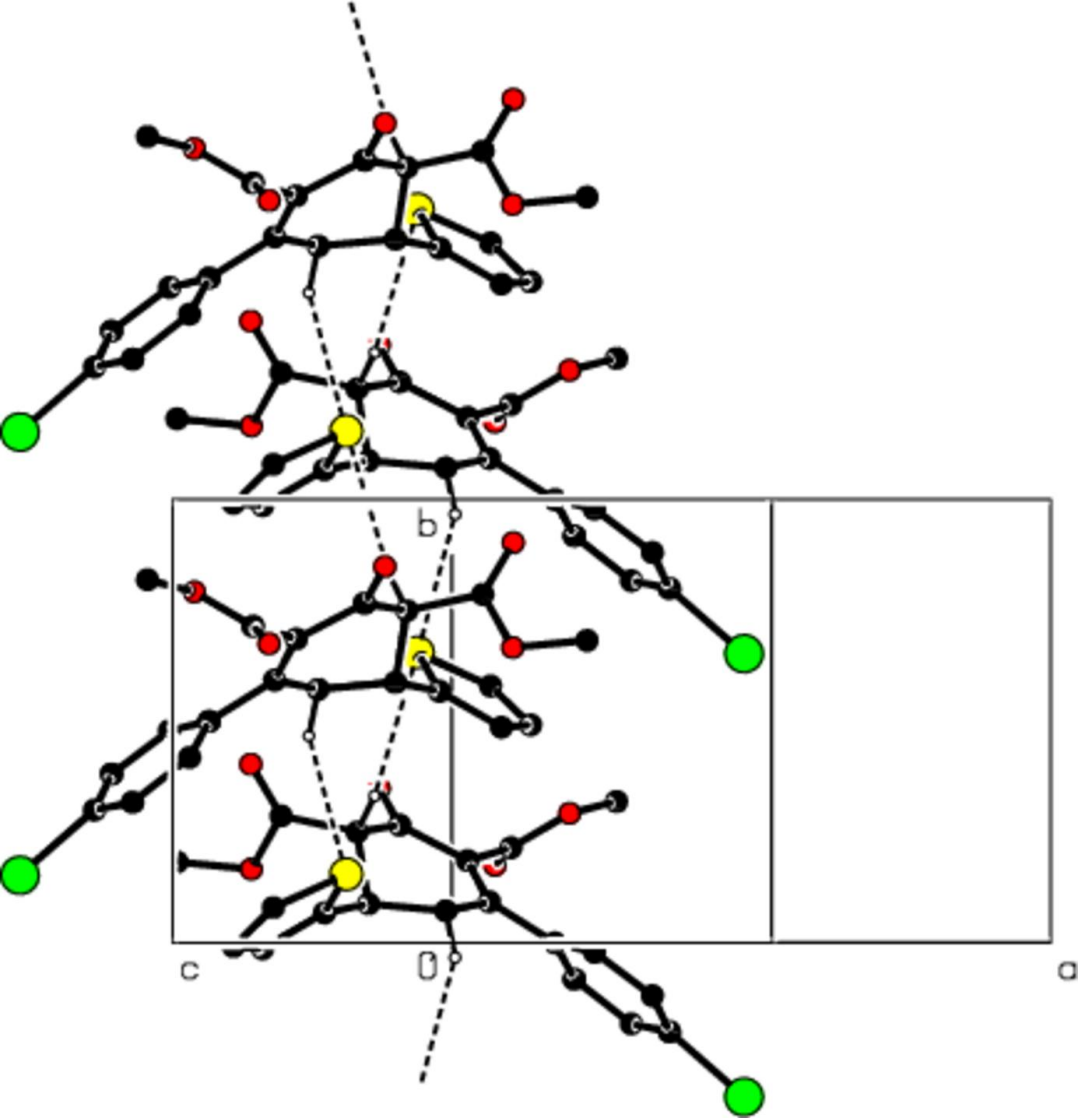
The packing viewed along the *b* axis of the title compound with C—H⋯S hydrogen bonds shown as dashed lines.

**Figure 5:**
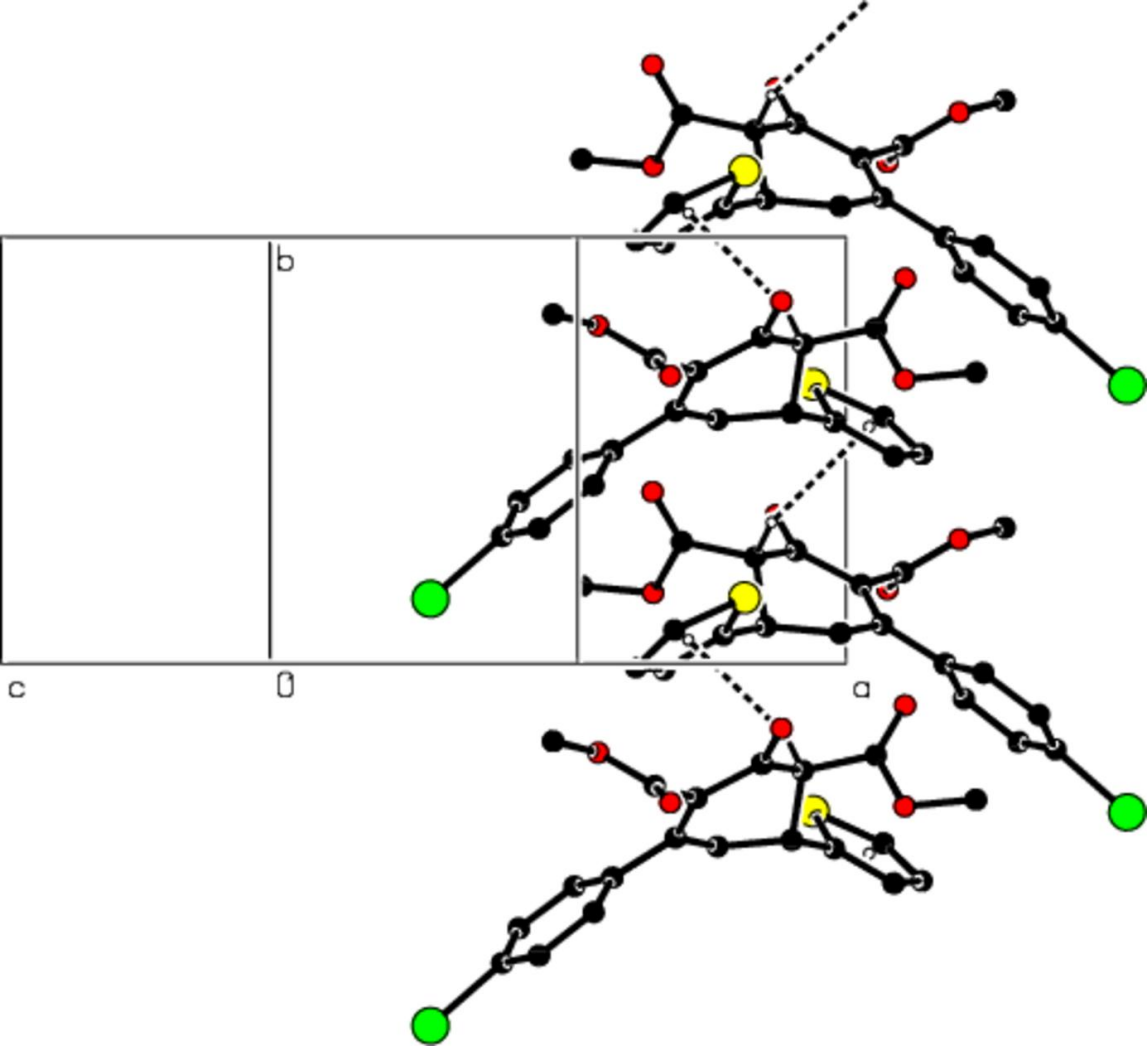
A view of the packing along the *b* axis of the title compound with C—H⋯π inter­actions shown as dashed lines.

**Table 1:** Hydrogen-bond geometry (Å, °) *Cg*1 and *Cg*2 are the centroids of the major (S21/C17–C20) and minor (S21*A*/C17*A*–C20*A*) disordered components of the thio­phene ring, respectively.

*D*—H⋯*A*	*D*—H	H⋯*A*	*D*⋯*A*	*D*—H⋯*A*
C3—H3⋯S21^i^	1.00	2.86	3.6775 (16)	139
C5—H5*B*⋯S21^ii^	0.99	2.84	3.5984 (15)	134
C3—H3⋯*Cg*1^i^	1.00	2.75	3.611 (2)	144
C3—H3⋯*Cg*2^i^	1.00	2.86	3.721 (11)	145
C4*A*—H4*A*⋯*Cg*1^i^	1.00	2.97	3.839 (8)	146

Symmetry codes: (i) 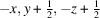 ; (ii) 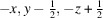 .

**Table 2:** Experimental details

Crystal data	
Chemical formula	C_20_H_17_BrO_5_S
*M*_r_	449.30
Crystal system, space group	Monoclinic, *P*2_1_/*c*
Temperature (K)	100
*a*, *b*, *c* (Å)	11.4670 (2), 8.4852 (2), 20.4823 (4)
β (°)	105.135 (2)
*V* (Å^3^)	1923.80 (7)
*Z*	4
Radiation type	Mo *K*α
μ (mm^−1^)	2.27
Crystal size (mm)	0.17 × 0.15 × 0.13

Data collection	
Diffractometer	XtaLAB Synergy, Dualflex, HyPix
Absorption correction	Gaussian (*CrysAlis PRO*; Rigaku OD, 2022 ▸)
*T*_min_, *T*_max_	0.705, 0.749
No. of measured, independent and observed [*I* > 2σ(*I*)] reflections	36460, 6958, 5872
*R*_int_	0.031
(sin θ/λ)_max_ (Å^−1^)	0.756

Refinement	
*R*[*F* ^2^ > 2σ(*F* ^2^)], *wR*(*F* ^2^), *S*	0.034, 0.086, 1.02
No. of reflections	6958
No. of parameters	281
No. of restraints	41
H-atom treatment	H-atom parameters constrained
Δρ_max_, Δρ_min_ (e Å^−3^)	0.67, −0.97

Computer programs: *CrysAlis PRO* (Rigaku OD, 2022 ▸), *SHELXT2014/5* (Sheldrick, 2015*a* ▸), *SHELXL2019/3* (Sheldrick, 2015*b* ▸), *ORTEP-3 for Windows* (Farrugia, 2012 ▸) and *PLATON* (Spek, 2020 ▸).

## supplementary crystallographic information

### Crystal data

**Table:**

C_20_H_17_BrO_5_S	*F*(000) = 912
*M**_r_* = 449.30	*D*_x_ = 1.551 Mg m^−^^3^
Monoclinic, *P*2_1_/*c*	Mo *K*α radiation, λ = 0.71073 Å
*a* = 11.4670 (2) Å	Cell parameters from 16653 reflections
*b* = 8.4852 (2) Å	θ = 2.4–34.9°
*c* = 20.4823 (4) Å	µ = 2.27 mm^−^^1^
β = 105.135 (2)°	*T* = 100 K
*V* = 1923.80 (7) Å^3^	Prism, colorless
*Z* = 4	0.17 × 0.15 × 0.13 mm

### Data collection

**Table:**

XtaLAB Synergy, Dualflex, HyPix diffractometer	6958 independent reflections
Radiation source: micro-focus sealed X-ray tube, PhotonJet (Mo) X-ray Source	5872 reflections with *I* > 2σ(*I*)
Mirror monochromator	*R*_int_ = 0.031
Detector resolution: 10.0000 pixels mm^-1^	θ_max_ = 32.5°, θ_min_ = 2.4°
ω scans	*h* = −17→17
Absorption correction: gaussian (CrysAlisPro; Rigaku OD, 2022)	*k* = −12→11
*T*_min_ = 0.705, *T*_max_ = 0.749	*l* = −30→30
36460 measured reflections	

### Refinement

**Table:**

Refinement on *F*^2^	41 restraints
Least-squares matrix: full	Hydrogen site location: inferred from neighbouring sites
*R*[*F*^2^ > 2σ(*F*^2^)] = 0.034	H-atom parameters constrained
*wR*(*F*^2^) = 0.086	*w* = 1/[σ^2^(*F*_o_^2^) + (0.0407*P*)^2^ + 1.251*P*] where *P* = (*F*_o_^2^ + 2*F*_c_^2^)/3
*S* = 1.02	(Δ/σ)_max_ = 0.002
6958 reflections	Δρ_max_ = 0.67 e Å^−^^3^
281 parameters	Δρ_min_ = −0.97 e Å^−^^3^

### Special details

**Table:**

Geometry. All esds (except the esd in the dihedral angle between two l.s. planes) are estimated using the full covariance matrix. The cell esds are taken into account individually in the estimation of esds in distances, angles and torsion angles; correlations between esds in cell parameters are only used when they are defined by crystal symmetry. An approximate (isotropic) treatment of cell esds is used for estimating esds involving l.s. planes.

### Fractional atomic coordinates and isotropic or equivalent isotropic displacement parameters (Å^2^)

**Table:**

	*x*	*y*	*z*	*U*_iso_*/*U*_eq_	Occ. (<1)
C1	0.06474 (12)	1.18537 (16)	0.08218 (7)	0.0143 (2)	
C2	−0.04850 (13)	1.26575 (16)	0.08357 (7)	0.0167 (2)	
C6	0.12509 (12)	1.09258 (16)	0.13373 (7)	0.0142 (2)	
C7	0.23349 (12)	0.99981 (16)	0.13173 (7)	0.0155 (2)	
C8	0.23883 (12)	0.91825 (17)	0.07335 (7)	0.0175 (2)	
H8	0.176356	0.932237	0.032951	0.021*	
C9	0.33408 (13)	0.81698 (18)	0.07342 (8)	0.0196 (3)	
H9	0.336538	0.760804	0.033686	0.024*	
C10	0.42545 (13)	0.79933 (19)	0.13246 (8)	0.0208 (3)	
C11	0.42406 (14)	0.8806 (2)	0.19092 (8)	0.0263 (3)	
H11	0.488418	0.869172	0.230650	0.032*	
C12	0.32712 (13)	0.9789 (2)	0.19054 (7)	0.0230 (3)	
H12	0.324273	1.032709	0.230758	0.028*	
C13	0.10805 (12)	1.21450 (17)	0.02019 (7)	0.0164 (2)	
C14	0.26561 (15)	1.3190 (2)	−0.02030 (9)	0.0273 (3)	
H14A	0.286093	1.217725	−0.037467	0.041*	
H14B	0.338866	1.382947	−0.005128	0.041*	
H14C	0.207112	1.374694	−0.056372	0.041*	
O2	−0.10404 (10)	1.34867 (12)	0.03704 (5)	0.0198 (2)	
O13	0.05503 (11)	1.17481 (14)	−0.03623 (6)	0.0227 (2)	
O14	0.21353 (9)	1.29185 (14)	0.03594 (5)	0.0205 (2)	
Br1	0.55053 (2)	0.65226 (2)	0.13401 (2)	0.02917 (6)	
C3	−0.08897 (13)	1.24975 (18)	0.14839 (7)	0.0117 (3)	0.8378 (15)
H3	−0.044629	1.331437	0.180640	0.014*	0.8378 (15)
C4	−0.05428 (13)	1.08776 (18)	0.18192 (7)	0.0109 (3)	0.8378 (15)
H4	−0.092206	1.004074	0.148783	0.013*	0.8378 (15)
C5	0.08168 (12)	1.07204 (16)	0.19659 (7)	0.0155 (2)	0.8378 (15)
H5A	0.120471	1.152304	0.230383	0.019*	0.8378 (15)
H5B	0.106284	0.966797	0.216259	0.019*	0.8378 (15)
C15	−0.22220 (17)	1.2862 (3)	0.13463 (12)	0.0159 (4)	0.8378 (15)
C16	−0.41712 (19)	1.1824 (4)	0.08947 (15)	0.0355 (5)	0.8378 (15)
H16A	−0.436708	1.189279	0.133177	0.053*	0.8378 (15)
H16B	−0.457703	1.090568	0.064505	0.053*	0.8378 (15)
H16C	−0.444531	1.278231	0.063267	0.053*	0.8378 (15)
O15	−0.2646 (4)	1.4029 (4)	0.1525 (2)	0.0261 (4)	0.8378 (15)
O16	−0.28797 (13)	1.16616 (18)	0.10046 (8)	0.0213 (3)	0.8378 (15)
C17	−0.0984 (3)	1.0674 (4)	0.24492 (16)	0.0176 (5)	0.8378 (15)
C18	−0.1998 (2)	0.9863 (3)	0.24920 (14)	0.0223 (5)	0.8378 (15)
H18	−0.252394	0.933027	0.212255	0.027*	0.8378 (15)
C19	−0.2169 (4)	0.9916 (5)	0.31673 (15)	0.0236 (6)	0.8378 (15)
H19	−0.280966	0.940071	0.329670	0.028*	0.8378 (15)
C20	−0.1304 (4)	1.0793 (6)	0.3595 (2)	0.0204 (6)	0.8378 (15)
H20	−0.127736	1.096845	0.405626	0.024*	0.8378 (15)
S21	−0.02657 (5)	1.15473 (6)	0.32094 (3)	0.02109 (12)	0.8378 (15)
C3A	−0.1240 (6)	1.1730 (9)	0.1263 (4)	0.0117 (3)	0.1622 (15)
H3A	−0.141139	1.063744	0.108021	0.014*	0.1622 (15)
C4A	−0.0380 (6)	1.1662 (10)	0.1961 (4)	0.0109 (3)	0.1622 (15)
H4A	−0.015521	1.276365	0.211844	0.013*	0.1622 (15)
C5A	0.08168 (12)	1.07204 (16)	0.19659 (7)	0.0155 (2)	0.1622 (15)
H5A1	0.146511	1.106604	0.236122	0.019*	0.1622 (15)
H5A2	0.067279	0.958518	0.202476	0.019*	0.1622 (15)
C15A	−0.2408 (10)	1.2545 (15)	0.1258 (8)	0.0159 (4)	0.1622 (15)
C16A	−0.4489 (10)	1.221 (2)	0.0924 (9)	0.0355 (5)	0.1622 (15)
H16D	−0.458095	1.231598	0.138416	0.053*	0.1622 (15)
H16E	−0.509847	1.148255	0.066583	0.053*	0.1622 (15)
H16F	−0.459580	1.324873	0.070295	0.053*	0.1622 (15)
O15A	−0.254 (2)	1.381 (2)	0.1486 (15)	0.0261 (4)	0.1622 (15)
O16A	−0.3313 (7)	1.1628 (11)	0.0950 (5)	0.0213 (3)	0.1622 (15)
C17A	−0.1017 (16)	1.090 (3)	0.2441 (8)	0.0176 (5)	0.1622 (15)
C18A	−0.0588 (12)	1.1351 (17)	0.3090 (6)	0.0223 (5)	0.1622 (15)
H18A	0.012306	1.197668	0.320826	0.027*	0.1622 (15)
C19A	−0.115 (3)	1.094 (4)	0.3598 (14)	0.0236 (6)	0.1622 (15)
H19A	−0.091317	1.117112	0.406784	0.028*	0.1622 (15)
C20A	−0.210 (3)	1.014 (3)	0.3255 (7)	0.0204 (6)	0.1622 (15)
H20A	−0.266675	0.974687	0.347789	0.024*	0.1622 (15)
S21A	−0.2302 (3)	0.9801 (5)	0.2404 (2)	0.02109 (12)	0.1622 (15)

### Atomic displacement parameters (Å^2^)

**Table:**

	*U*^11^	*U*^22^	*U*^33^	*U*^12^	*U*^13^	*U*^23^
C1	0.0177 (5)	0.0133 (6)	0.0129 (5)	0.0007 (4)	0.0058 (4)	−0.0008 (4)
C2	0.0214 (6)	0.0149 (6)	0.0150 (6)	0.0040 (5)	0.0069 (5)	0.0014 (5)
C6	0.0155 (5)	0.0145 (6)	0.0128 (5)	0.0002 (4)	0.0039 (4)	−0.0013 (4)
C7	0.0157 (5)	0.0176 (6)	0.0138 (6)	0.0018 (4)	0.0049 (4)	0.0007 (5)
C8	0.0191 (6)	0.0183 (6)	0.0145 (6)	0.0039 (5)	0.0033 (5)	0.0001 (5)
C9	0.0229 (6)	0.0211 (7)	0.0161 (6)	0.0064 (5)	0.0073 (5)	0.0013 (5)
C10	0.0193 (6)	0.0270 (7)	0.0182 (6)	0.0091 (5)	0.0088 (5)	0.0053 (5)
C11	0.0198 (6)	0.0409 (9)	0.0169 (7)	0.0106 (6)	0.0024 (5)	−0.0010 (6)
C12	0.0194 (6)	0.0344 (8)	0.0142 (6)	0.0071 (6)	0.0024 (5)	−0.0043 (6)
C13	0.0190 (6)	0.0159 (6)	0.0158 (6)	0.0040 (5)	0.0072 (5)	0.0026 (5)
C14	0.0253 (7)	0.0346 (9)	0.0273 (8)	0.0022 (6)	0.0161 (6)	0.0083 (6)
O2	0.0244 (5)	0.0200 (5)	0.0152 (5)	0.0070 (4)	0.0057 (4)	0.0038 (4)
O13	0.0278 (5)	0.0271 (6)	0.0142 (5)	−0.0001 (4)	0.0072 (4)	0.0006 (4)
O14	0.0188 (4)	0.0256 (5)	0.0192 (5)	0.0000 (4)	0.0087 (4)	0.0035 (4)
Br1	0.02706 (8)	0.04121 (11)	0.02210 (8)	0.02021 (7)	0.01151 (6)	0.00851 (6)
C3	0.0137 (6)	0.0113 (6)	0.0104 (6)	0.0023 (5)	0.0039 (5)	0.0003 (5)
C4	0.0145 (6)	0.0082 (7)	0.0100 (6)	0.0012 (5)	0.0033 (5)	−0.0005 (5)
C5	0.0175 (5)	0.0169 (6)	0.0127 (5)	0.0040 (4)	0.0051 (4)	0.0015 (4)
C15	0.0163 (8)	0.0193 (11)	0.0123 (9)	0.0022 (6)	0.0041 (7)	0.0025 (7)
C16	0.0152 (10)	0.0528 (17)	0.0369 (11)	0.0001 (9)	0.0042 (9)	0.0064 (11)
O15	0.0242 (11)	0.0276 (13)	0.0281 (9)	0.0128 (8)	0.0095 (7)	−0.0011 (9)
O16	0.0096 (5)	0.0294 (6)	0.0230 (6)	−0.0016 (6)	0.0010 (6)	0.0006 (5)
C17	0.0192 (6)	0.0201 (14)	0.0140 (6)	0.0052 (8)	0.0054 (5)	0.0051 (7)
C18	0.0257 (12)	0.0247 (10)	0.0176 (10)	0.0025 (10)	0.0079 (9)	0.0030 (8)
C19	0.0234 (9)	0.0268 (18)	0.0219 (12)	−0.0025 (11)	0.0080 (11)	0.0045 (9)
C20	0.0279 (18)	0.0194 (15)	0.0170 (8)	0.0046 (11)	0.0116 (10)	0.0022 (8)
S21	0.0263 (3)	0.0198 (2)	0.0179 (2)	0.00020 (18)	0.00703 (18)	0.00161 (17)
C3A	0.0137 (6)	0.0113 (6)	0.0104 (6)	0.0023 (5)	0.0039 (5)	0.0003 (5)
C4A	0.0145 (6)	0.0082 (7)	0.0100 (6)	0.0012 (5)	0.0033 (5)	−0.0005 (5)
C5A	0.0175 (5)	0.0169 (6)	0.0127 (5)	0.0040 (4)	0.0051 (4)	0.0015 (4)
C15A	0.0163 (8)	0.0193 (11)	0.0123 (9)	0.0022 (6)	0.0041 (7)	0.0025 (7)
C16A	0.0152 (10)	0.0528 (17)	0.0369 (11)	0.0001 (9)	0.0042 (9)	0.0064 (11)
O15A	0.0242 (11)	0.0276 (13)	0.0281 (9)	0.0128 (8)	0.0095 (7)	−0.0011 (9)
O16A	0.0096 (5)	0.0294 (6)	0.0230 (6)	−0.0016 (6)	0.0010 (6)	0.0006 (5)
C17A	0.0192 (6)	0.0201 (14)	0.0140 (6)	0.0052 (8)	0.0054 (5)	0.0051 (7)
C18A	0.0257 (12)	0.0247 (10)	0.0176 (10)	0.0025 (10)	0.0079 (9)	0.0030 (8)
C19A	0.0234 (9)	0.0268 (18)	0.0219 (12)	−0.0025 (11)	0.0080 (11)	0.0045 (9)
C20A	0.0279 (18)	0.0194 (15)	0.0170 (8)	0.0046 (11)	0.0116 (10)	0.0022 (8)
S21A	0.0263 (3)	0.0198 (2)	0.0179 (2)	0.00020 (18)	0.00703 (18)	0.00161 (17)

### Geometric parameters (Å, º)

**Table:**

C1—C6	1.3536 (18)	C15—O16	1.349 (2)
C1—C2	1.4736 (19)	C16—O16	1.445 (2)
C1—C13	1.4997 (19)	C16—H16A	0.9800
C2—O2	1.2214 (17)	C16—H16B	0.9800
C2—C3	1.523 (2)	C16—H16C	0.9800
C2—C3A	1.590 (7)	C17—C18	1.374 (4)
C6—C7	1.4808 (19)	C17—S21	1.727 (3)
C6—C5A	1.5067 (19)	C18—C19	1.448 (4)
C6—C5	1.5067 (19)	C18—H18	0.9500
C7—C8	1.3967 (19)	C19—C20	1.360 (3)
C7—C12	1.4000 (19)	C19—H19	0.9500
C8—C9	1.3894 (19)	C20—S21	1.714 (3)
C8—H8	0.9500	C20—H20	0.9500
C9—C10	1.386 (2)	C3A—C15A	1.506 (11)
C9—H9	0.9500	C3A—C4A	1.511 (9)
C10—C11	1.385 (2)	C3A—H3A	1.0000
C10—Br1	1.8952 (14)	C4A—C17A	1.515 (11)
C11—C12	1.388 (2)	C4A—C5A	1.586 (7)
C11—H11	0.9500	C4A—H4A	1.0000
C12—H12	0.9500	C5A—H5A1	0.9900
C13—O13	1.2048 (18)	C5A—H5A2	0.9900
C13—O14	1.3395 (18)	C15A—O15A	1.192 (12)
C14—O14	1.4477 (19)	C15A—O16A	1.318 (10)
C14—H14A	0.9800	C16A—O16A	1.425 (11)
C14—H14B	0.9800	C16A—H16D	0.9800
C14—H14C	0.9800	C16A—H16E	0.9800
C3—C15	1.511 (2)	C16A—H16F	0.9800
C3—C4	1.542 (2)	C17A—C18A	1.346 (13)
C3—H3	1.0000	C17A—S21A	1.728 (13)
C4—C17	1.514 (3)	C18A—C19A	1.407 (14)
C4—C5	1.5146 (19)	C18A—H18A	0.9500
C4—H4	1.0000	C19A—C20A	1.313 (17)
C5—H5A	0.9900	C19A—H19A	0.9500
C5—H5B	0.9900	C20A—S21A	1.721 (12)
C15—O15	1.201 (2)	C20A—H20A	0.9500
			
C6—C1—C2	121.86 (12)	O16—C16—H16A	109.5
C6—C1—C13	122.78 (12)	O16—C16—H16B	109.5
C2—C1—C13	115.36 (11)	H16A—C16—H16B	109.5
O2—C2—C1	122.29 (13)	O16—C16—H16C	109.5
O2—C2—C3	121.07 (13)	H16A—C16—H16C	109.5
C1—C2—C3	116.51 (12)	H16B—C16—H16C	109.5
O2—C2—C3A	117.8 (3)	C15—O16—C16	114.84 (18)
C1—C2—C3A	113.0 (3)	C18—C17—C4	126.0 (3)
C1—C6—C7	123.33 (12)	C18—C17—S21	111.7 (2)
C1—C6—C5A	121.20 (12)	C4—C17—S21	122.21 (19)
C7—C6—C5A	115.42 (11)	C17—C18—C19	111.9 (3)
C1—C6—C5	121.20 (12)	C17—C18—H18	124.0
C7—C6—C5	115.42 (11)	C19—C18—H18	124.0
C8—C7—C12	118.58 (13)	C20—C19—C18	111.9 (4)
C8—C7—C6	120.79 (12)	C20—C19—H19	124.0
C12—C7—C6	120.29 (12)	C18—C19—H19	124.0
C9—C8—C7	121.04 (13)	C19—C20—S21	112.7 (4)
C9—C8—H8	119.5	C19—C20—H20	123.7
C7—C8—H8	119.5	S21—C20—H20	123.7
C10—C9—C8	118.87 (14)	C20—S21—C17	91.71 (18)
C10—C9—H9	120.6	C15A—C3A—C4A	112.6 (8)
C8—C9—H9	120.6	C15A—C3A—C2	112.3 (8)
C11—C10—C9	121.58 (13)	C4A—C3A—C2	103.0 (5)
C11—C10—Br1	119.51 (11)	C15A—C3A—H3A	109.6
C9—C10—Br1	118.83 (12)	C4A—C3A—H3A	109.6
C10—C11—C12	118.95 (14)	C2—C3A—H3A	109.6
C10—C11—H11	120.5	C3A—C4A—C17A	108.9 (9)
C12—C11—H11	120.5	C3A—C4A—C5A	112.0 (5)
C11—C12—C7	120.95 (14)	C17A—C4A—C5A	110.1 (10)
C11—C12—H12	119.5	C3A—C4A—H4A	108.6
C7—C12—H12	119.5	C17A—C4A—H4A	108.6
O13—C13—O14	124.46 (13)	C5A—C4A—H4A	108.6
O13—C13—C1	124.77 (13)	C6—C5A—C4A	114.5 (3)
O14—C13—C1	110.77 (12)	C6—C5A—H5A1	108.6
O14—C14—H14A	109.5	C4A—C5A—H5A1	108.6
O14—C14—H14B	109.5	C6—C5A—H5A2	108.6
H14A—C14—H14B	109.5	C4A—C5A—H5A2	108.6
O14—C14—H14C	109.5	H5A1—C5A—H5A2	107.6
H14A—C14—H14C	109.5	O15A—C15A—O16A	123.8 (16)
H14B—C14—H14C	109.5	O15A—C15A—C3A	127.6 (15)
C13—O14—C14	114.86 (12)	O16A—C15A—C3A	108.7 (9)
C15—C3—C2	110.03 (14)	O16A—C16A—H16D	109.5
C15—C3—C4	113.35 (14)	O16A—C16A—H16E	109.5
C2—C3—C4	111.47 (12)	H16D—C16A—H16E	109.5
C15—C3—H3	107.2	O16A—C16A—H16F	109.5
C2—C3—H3	107.2	H16D—C16A—H16F	109.5
C4—C3—H3	107.2	H16E—C16A—H16F	109.5
C17—C4—C5	112.12 (17)	C15A—O16A—C16A	115.5 (11)
C17—C4—C3	112.12 (17)	C18A—C17A—C4A	113.8 (11)
C5—C4—C3	107.42 (12)	C18A—C17A—S21A	106.6 (9)
C17—C4—H4	108.3	C4A—C17A—S21A	138.8 (12)
C5—C4—H4	108.3	C17A—C18A—C19A	122.6 (18)
C3—C4—H4	108.3	C17A—C18A—H18A	118.7
C6—C5—C4	111.90 (11)	C19A—C18A—H18A	118.7
C6—C5—H5A	109.2	C20A—C19A—C18A	102 (3)
C4—C5—H5A	109.2	C20A—C19A—H19A	129.0
C6—C5—H5B	109.2	C18A—C19A—H19A	129.0
C4—C5—H5B	109.2	C19A—C20A—S21A	120 (2)
H5A—C5—H5B	107.9	C19A—C20A—H20A	119.9
O15—C15—O16	124.4 (3)	S21A—C20A—H20A	119.9
O15—C15—C3	125.5 (3)	C20A—S21A—C17A	88.6 (12)
O16—C15—C3	110.05 (16)		
			
C6—C1—C2—O2	−179.40 (14)	C2—C3—C15—O15	−107.7 (4)
C13—C1—C2—O2	0.9 (2)	C4—C3—C15—O15	126.7 (4)
C6—C1—C2—C3	4.7 (2)	C2—C3—C15—O16	73.8 (2)
C13—C1—C2—C3	−174.94 (12)	C4—C3—C15—O16	−51.8 (2)
C6—C1—C2—C3A	−29.3 (3)	O15—C15—O16—C16	−3.4 (5)
C13—C1—C2—C3A	151.1 (3)	C3—C15—O16—C16	175.03 (18)
C2—C1—C6—C7	174.86 (13)	C5—C4—C17—C18	−139.1 (3)
C13—C1—C6—C7	−5.5 (2)	C3—C4—C17—C18	100.0 (3)
C2—C1—C6—C5A	−2.4 (2)	C5—C4—C17—S21	43.8 (3)
C13—C1—C6—C5A	177.20 (12)	C3—C4—C17—S21	−77.1 (3)
C2—C1—C6—C5	−2.4 (2)	C4—C17—C18—C19	−179.2 (4)
C13—C1—C6—C5	177.20 (12)	S21—C17—C18—C19	−1.8 (3)
C1—C6—C7—C8	−42.0 (2)	C17—C18—C19—C20	1.7 (4)
C5A—C6—C7—C8	135.42 (14)	C18—C19—C20—S21	−0.7 (4)
C5—C6—C7—C8	135.42 (14)	C19—C20—S21—C17	−0.3 (3)
C1—C6—C7—C12	144.72 (15)	C18—C17—S21—C20	1.2 (2)
C5A—C6—C7—C12	−37.84 (19)	C4—C17—S21—C20	178.7 (4)
C5—C6—C7—C12	−37.84 (19)	O2—C2—C3A—C15A	−26.9 (8)
C12—C7—C8—C9	0.7 (2)	C1—C2—C3A—C15A	−178.5 (6)
C6—C7—C8—C9	−172.68 (14)	O2—C2—C3A—C4A	−148.3 (4)
C7—C8—C9—C10	−0.9 (2)	C1—C2—C3A—C4A	60.1 (5)
C8—C9—C10—C11	−0.3 (2)	C15A—C3A—C4A—C17A	54.9 (13)
C8—C9—C10—Br1	176.45 (12)	C2—C3A—C4A—C17A	176.1 (10)
C9—C10—C11—C12	1.6 (3)	C15A—C3A—C4A—C5A	176.9 (7)
Br1—C10—C11—C12	−175.12 (14)	C2—C3A—C4A—C5A	−61.9 (6)
C10—C11—C12—C7	−1.8 (3)	C1—C6—C5A—C4A	0.2 (4)
C8—C7—C12—C11	0.7 (2)	C7—C6—C5A—C4A	−177.3 (3)
C6—C7—C12—C11	174.06 (15)	C3A—C4A—C5A—C6	35.3 (7)
C6—C1—C13—O13	116.12 (17)	C17A—C4A—C5A—C6	156.6 (8)
C2—C1—C13—O13	−64.22 (19)	C4A—C3A—C15A—O15A	52 (3)
C6—C1—C13—O14	−64.33 (17)	C2—C3A—C15A—O15A	−64 (3)
C2—C1—C13—O14	115.33 (13)	C4A—C3A—C15A—O16A	−128.5 (11)
O13—C13—O14—C14	−3.2 (2)	C2—C3A—C15A—O16A	115.7 (11)
C1—C13—O14—C14	177.23 (12)	O15A—C15A—O16A—C16A	−2 (3)
O2—C2—C3—C15	23.8 (2)	C3A—C15A—O16A—C16A	177.9 (11)
C1—C2—C3—C15	−160.24 (13)	C3A—C4A—C17A—C18A	−153.1 (13)
O2—C2—C3—C4	150.48 (14)	C5A—C4A—C17A—C18A	83.8 (16)
C1—C2—C3—C4	−33.59 (18)	C3A—C4A—C17A—S21A	15 (3)
C15—C3—C4—C17	−52.9 (2)	C5A—C4A—C17A—S21A	−109 (2)
C2—C3—C4—C17	−177.67 (17)	C4A—C17A—C18A—C19A	172 (2)
C15—C3—C4—C5	−176.48 (14)	S21A—C17A—C18A—C19A	0.2 (18)
C2—C3—C4—C5	58.71 (15)	C17A—C18A—C19A—C20A	−2 (3)
C1—C6—C5—C4	29.70 (18)	C18A—C19A—C20A—S21A	3 (3)
C7—C6—C5—C4	−147.81 (12)	C19A—C20A—S21A—C17A	−2 (2)
C17—C4—C5—C6	−179.92 (17)	C18A—C17A—S21A—C20A	1.0 (11)
C3—C4—C5—C6	−56.31 (15)	C4A—C17A—S21A—C20A	−167 (3)

### Hydrogen-bond geometry (Å, º)

*Cg*1 and *Cg*2 are the centroids of the major (S21/C17–C20) and minor (S21*A*/C17*A*–C20*A*) disordered components of the thiophene ring, respectively.

**Table:**

*D*—H···*A*	*D*—H	H···*A*	*D*···*A*	*D*—H···*A*
C3—H3···S21^i^	1.00	2.86	3.6775 (16)	139
C5—H5*A*···S21	0.99	2.81	3.1892 (15)	103
C5—H5*B*···S21^ii^	0.99	2.84	3.5984 (15)	134
C3—H3···*Cg*1^i^	1.00	2.75	3.611 (2)	144
C3—H3···*Cg*2^i^	1.00	2.86	3.721 (11)	145
C4*A*—H4*A*···*Cg*1^i^	1.00	2.97	3.839 (8)	146

Symmetry codes: (i) −*x*, *y*+1/2, −*z*+1/2; (ii) −*x*, *y*−1/2, −*z*+1/2.
